# From BBB to PPP: Bioenergetic requirements and challenges for oligodendrocytes in health and disease

**DOI:** 10.1111/jnc.16219

**Published:** 2024-09-10

**Authors:** Milton Guilherme Forestieri Fernandes, Florian Pernin, Jack P. Antel, Timothy E. Kennedy

**Affiliations:** ^1^ Neuroimmunological Diseases and Glia Biology Research Group, Department of Neurology and Neurosurgery, Montreal Neurological Institute McGill University Montreal Quebec Canada

**Keywords:** hypoperfusion, metabolic stress, metabolism, multiple sclerosis, myelin, oligodendrocyte injury

## Abstract

Mature myelinating oligodendrocytes, the cells that produce the myelin sheath that insulates axons in the central nervous system, have distinct energetic and metabolic requirements compared to neurons. Neurons require substantial energy to execute action potentials, while the energy needs of oligodendrocytes are directed toward building the lipid‐rich components of myelin and supporting neuronal metabolism by transferring glycolytic products to axons as additional fuel. The utilization of energy metabolites in the brain parenchyma is tightly regulated to meet the needs of different cell types. Disruption of the supply of metabolites can lead to stress and oligodendrocyte injury, contributing to various neurological disorders, including some demyelinating diseases. Understanding the physiological properties, structures, and mechanisms involved in oligodendrocyte energy metabolism, as well as the relationship between oligodendrocytes and neighboring cells, is crucial to investigate the underlying pathophysiology caused by metabolic impairment in these disorders. In this review, we describe the particular physiological properties of oligodendrocyte energy metabolism and the response of oligodendrocytes to metabolic stress. We delineate the relationship between oligodendrocytes and other cells in the context of the neurovascular unit, and the regulation of metabolite supply according to energetic needs. We focus on the specific bioenergetic requirements of oligodendrocytes and address the disruption of metabolic energy in demyelinating diseases. We encourage further studies to increase understanding of the significance of metabolic stress on oligodendrocyte injury, to support the development of novel therapeutic approaches for the treatment of demyelinating diseases.

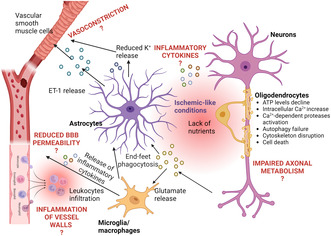

## Graphical abstract

Mature myelinating oligodendrocytes have distinct energetic and metabolic requirements compared to neurons. In demyelinating diseases, metabolic stress is a potential cause of oligodendrocyte injury. To understand the pathophysiology that results from metabolic impairments in these disorders it is crucial to investigate oligodendrocyte energy metabolism and the relationship between oligodendrocytes and neighboring cells, in particular the cells that make up the neurovascular unit. Here, we review the properties of oligodendrocyte energy metabolism and address the disruption of metabolic energy in demyelinating diseases. Understanding these molecular mechanisms and cellular relationships aims to identify potential targets for treatments to promote remyelination and recovery.

**Abbreviations**
AGAT: L‐arginine: glycine amidinotransferase
AMPK: AMP‐activated protein kinase
BBB: blood–brain barrier
BCAS: bilateral carotid artery stenosis
CNS: central nervous system
CSCL1: chemokine C‐X‐C motif ligand 1
CXCL13: C‐X‐C motif chemokine ligand 13
EAE: experimental autoimmune encephalomyelitis
EIF2B: eukaryotic initiation factor 2 B
ET1: endothelin‐1
ETC: electron transport chain
FABP: fatty acid binding protein
FATP: fatty acid transport protein
FGF: fibroblast growth factor
GH: growth hormone
GLUT: glucose transporter
GP: glycogen phosphorylase
GPR81: G protein‐coupled receptor 81
GS: glycogen synthase
HCA1: hydroxycarboxylic acid receptor 1
HIF: hypoxia‐inducible factor
HIF1: hypoxia‐inducible factor 1
IFNγ: interferon gamma
IGF‐1: insulin‐like growth factor 1
IL10: interleukin 10
LD: lipid droplets
LDH: lactate dehydrogenase
MCP1: monocyte chemoattractant protein‐1
MCT: monocarboxylate transporter
MMP9: matrix metalloproteinase‐9
MS: multiple sclerosis
NAA: N‐acetyl aspartate
NADH: nicotinamide adenine dinucleotide (reduced)
NADPH: nicotinamide adenine dinucleotide phosphate (reduced)
NAWM: normal‐apparent white matter
NMDA: N‐methyl‐D‐aspartate
NVU: neurovascular unit
OPC: oligodendrocyte precursor cell
OXPHOS: oxidative phosphorylation
PDGF: platelet‐derived growth factor
PPP: pentose phosphate pathway
ROS: reactive oxygen species
SPMS: secondary progressive multiple sclerosis
TFG‐β: transforming growth factor‐beta
TNFα: tumor necrosis factor‐alpha
VEGF: vascular endothelial growth factor
VEGF‐A: vascular endothelial growth factor A
βHB: beta‐hydroxybutyrate

## 1 INTRODUCTION

Mature myelinating oligodendrocytes are large highly specialized post‐mitotic cells with distinct energetic and metabolic needs compared to their more illustrious partners in the CNS, neurons. Oligodendrocytes have much lower energetic demands than neurons, which require substantial energy to support the execution of action potentials (Rone et al., 2016; Rosko et al., 2019). Oligodendroglial energy demands are required to meet two of the main oligodendrocyte functions: myelination and providing metabolic support to neurons. A substantial portion of the energetic metabolites taken up by oligodendrocytes, such as glucose and ketone bodies, are used to build the specialized lipid‐rich structure of myelin (Sykes et al., 1986). Oligodendrocytes also support the energetic needs of neurons, being responsible, along with astrocytes, for supplying neurons with glycolytic products, lactate and pyruvate, providing extra fuel to maintain their intense activity (Fünfschilling et al., 2012; Saab et al., 2013; Späte et al., 2024).

A well‐orchestrated distribution of energetic metabolites between cells in the brain parenchyma depends on a series of regulatory mechanisms that adjust the supply of metabolic substrates to meet local energetic demand (Kaplan et al., 2020; Kugler et al., 2021). Impairment of this coupling or extrinsic factors that disturb the proper delivery of metabolites can cause cellular injuries that contribute to a range of neurologic disorders (Apátiga‐Pérez et al., 2022; Cai et al., 2017; Iadecola, 2017) that include demyelinating diseases (Chen et al., 2017; De Keyser et al., 2008; D'Haeseleer et al., 2011, 2013, 2015; Marshall et al., 2014).

In this review, we address the physiological properties, structures, and mechanisms involved in oligodendrocyte energy metabolism and their dynamic and delicate relationship with neighboring cells. We explore the impact that disruption of these mechanisms can have on oligodendrocyte function and the development of genetic and acquired demyelinating diseases. Studying these features yields opportunities to better understand these disorders and support the development of new treatment strategies.

## 2 ENERGY METABOLISM IN OLIGODENDROCYTES

Myelin maintenance requires significant amounts of ATP (Harris & Attwell, 2012), yet glycolytic metabolites are also essential precursors for the production of myelin lipids (Sánchez‐Abarca et al., 2001). The metabolic profile of mature oligodendrocytes is different from oligodendrocyte precursor cells (OPCs), which are migratory and mitotically active. Mature oligodendrocytes are less dynamic and less metabolically active than OPCs but still require energy and molecular precursors to support and maintain large myelinated internodes and shuttle glycolytic products to neurons (Philips & Rothstein, 2017; Rone et al., 2016; Späte et al., 2024). A major shift in metabolic profile during oligodendrocyte differentiation occurs in pre‐oligodendrocytes, in which myelin production is high.

Studies based on cells derived from rat pups indicate that most ATP in a mature oligodendrocyte is derived from glycolysis, while in OPCs, a greater contribution from mitochondrial oxidative phosphorylation (OXPHOS) via the tricarboxylic acid cycle (TCA) underlies ATP production (Rone et al., 2016). OPC to oligodendrocyte differentiation includes a reduction in ATP production. Metabolic flux assays using a Seahorse real‐time analyzer of cell metabolism revealed increased extracellular acidification by adult rat oligodendrocytes compared to OPCs, consistent with greater lactate release by mature oligodendrocytes (Rao et al., 2017). One mechanism that may contribute is OPC expression of G protein‐coupled receptor 17 (GPR17), which inhibits myelination and glycolysis. GPR17 is internalized and down‐regulated during the transition of OPCs to mature oligodendrocytes, leading to an increase in glycolysis coincident with myelin synthesis (Marangon et al., 2022). Future studies to more deeply understand the mechanisms involved in this metabolic shift from OPC to oligodendrocyte should be conducted, particularly in relation to how the utilization of OXPHOS to generate ATP is regulated.

## 3 IMPACT OF METABOLIC STRESS ON HUMAN OLIGODENDROCYTES

Oligodendroglial injury responses to metabolic stress, which we define as a cellular state triggered by low energy, reflect the distinct bioenergetic properties of these cells (Rone et al., 2016). When challenged with a metabolic stress, oligodendrocytes retract their processes, termed oligodendrogliopathy, and reduce energy utilization and ATP production before any signs of cell death (Rone et al., 2016). These findings suggest that when challenged by metabolic stress, as in acute and chronic MS lesions undergoing demyelination (Kuhlmann et al., 2017), oligodendrocytes are less capable of sustaining myelin, consistent with process retraction, and are also less able to release the products of glycolysis, lactate and pyruvate, that are required to support neurons (Rao et al., 2017).

The metabolic activity of OPCs and oligodendrocytes is notably lower in human cells than in rodent‐derived cells, and human oligodendrocytes, studied in cell culture, are surprisingly resistant to cell death. Treatment for 6 days with pro‐inflammatory cytokines (TNFα, IFNγ) or an excitotoxin (glutamate), that rapidly triggers apoptosis in rodent oligodendrocytes, did not increase human oligodendrocyte cell death (Rao et al., 2017). Human mature oligodendrocytes are also resilient to metabolic stress induced by culturing cells in nutrient‐deprived conditions (Pernin et al., 2022). In comparison, human OPCs are more susceptible to cell death in the same conditions, than are human mature oligodendrocytes, exhibiting a higher propensity to trigger apoptosis (Cui et al., 2013). Further, pediatric‐derived human oligodendrocytes are more susceptible to cell death triggered by metabolic stress compared to human adolescent or adult‐derived oligodendrocytes, indicating that the resistance of human mature oligodendrocytes to cell death increases with the age of the individual and stage of cellular maturation (Fernandes et al., 2021).

**FIGURE 1:**
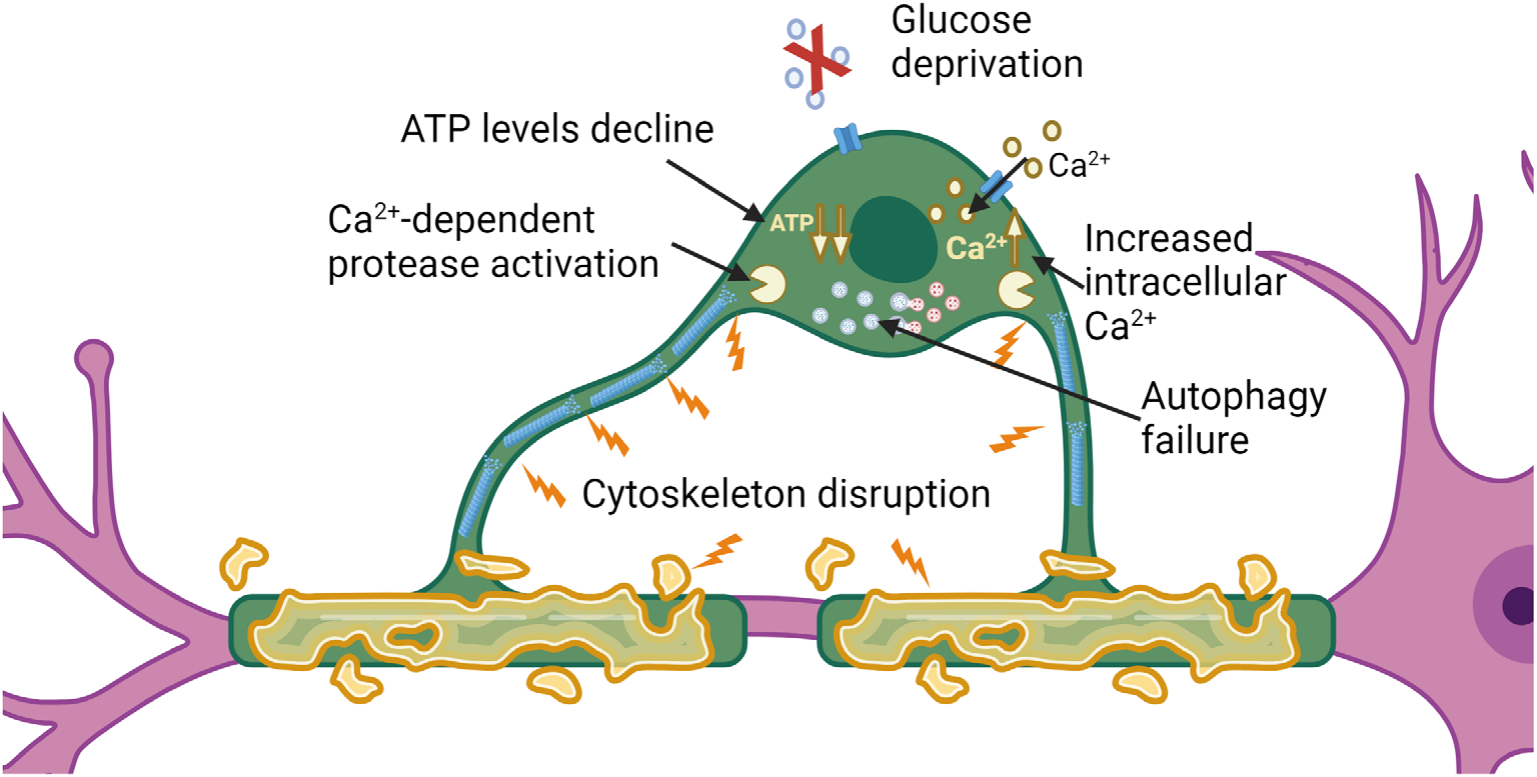
Oligodendrocyte injury mechanisms resulting from metabolic stress. Glucose deprivation causes failure of ATP production. As ATP decreases, the capacity to execute autophagy is lost and autophagosomes accumulate. ATP‐dependent plasma membrane pumps are compromised and the cell becomes incapable of pumping Ca^2+^ out of the cytosol. The increased intracellular Ca^2+^ concentration activates Ca^2+^‐dependent proteases leading to degradation. Failure of autophagy, which is also an ATP‐dependent process, further aggravates the process as cellular components can no longer be recycled as substrates for energy production. Created with BioRender.

Within the oligodendrocyte lineage, the increased resistance of human mature oligodendrocytes to apoptosis compared to OPCs reflects a relative increase in expression of anti‐apoptotic compared to pro‐apoptotic molecules (Fernandes et al., 2021). When investigating the underlying mechanism of cell death triggered by metabolic stress as a result of glucose deprivation, we found that human oligodendrocytes are resistant to ferroptosis and MPT‐driven necrosis. Cleavage of spectrin, a target of the Ca^2+^‐dependent protease calpain, was detected in human oligodendrocytes subjected to these stress conditions in cell culture. Calpain activation, as a result of increased intracellular Ca^2+^, likely reflects failure to maintain the activity required to pump of Ca^2+^ out of the cell, a process that consumes substantial ATP. This degradation is aggravated by autophagy failure, also likely resulting from the depletion of cellular ATP (Figure 1) (Fernandes et al., 2023). As cells shrink in this process, the fate of the cellular material that is lost is still not clear. It may be consumed by the cell in an attempt to generate ATP, or shed and taken up by other cells.

When ATP runs low, cells may also temporarily halt certain processes, for example, by essentially shelving newly translated proteins that may no longer be necessary in the context of cellular metabolic stress. By inhibiting anabolic pathways, such as protein translation, cellular energy resources can then be reallocated to pro‐survival strategies. This may engage the integrated stress response (ISR) and regulation of mechanistic target of rapamycin (mTOR), two protective mechanisms that maintain cellular proteostasis. Roles for these mechanisms have recently been demonstrated in human oligodendrocytes exposed to pro‐inflammatory and metabolic stress conditions (Pernin et al., 2022, 2024).

Global attenuation of protein production by ISR and mTOR signaling may ultimately lead to the formation of stress granules (SGs), micron‐sized phase‐separated ribonucleoprotein aggregates that promote cell survival by packaging mRNAs and translational machinery for temporary storage and safekeeping. Assembly and disassembly of SGs in the cytosol occurs by a highly regulated energy‐dependent process (Wang, Tian, et al., 2022). Conventional SG formation is initiated by eIF2A phosphorylation (ISR activation), leading to inhibition of new translation, polysome runoff, and release of free mRNAs. Recent studies of metabolic stress have highlighted the formation of energy deficiency‐induced granules (eSGs). These are distinguished from conventional granules based on their dynamics and mechanisms of assembly (Pernin et al., 2024; Wang, Tian, et al., 2022). Increasing evidence suggests that the composition of eSGs may be diverse and stress‐specific (Aulas et al., 2017; Markmiller et al., 2018).

SGs are dynamic and temporary, resolving with stress relief to reinitiate translation and promote cell survival (Reineke et al., 2018). The functional consequences of SG formation in the CNS are incompletely understood, and granule persistence has been linked to degenerative brain disorders, especially those that involve disruption of cellular energy metabolism (Pernin et al., 2024). As for other cell types, although SG formation in oligodendrocytes may initially be an adaptive pro‐survival response to cell stress, SG persistence shuts down protein synthesis, causes growth arrest, and results in cell death.

## 4 SUPPLY OF NUTRIENTS AND GROWTH FACTORS TO OLIGODENDROCYTES: THE NEUROVASCULAR UNIT (NVU)

The intense activity of the brain requires an enormous amount of energy, consuming an average of 200 g of glucose per day, which must be shared between oligodendrocytes and the other cells of the brain parenchyma. This consumption varies considerably, increasing by up to 12% in stressful situations and decreasing by ~40% during sleep (Boyle et al., 1994; Madsen et al., 1995; Peters, 2011; Reinmuth et al., 1965). To fulfill these energetic needs, the supply of nutrients must be precise in quantity, time, and location. To accomplish this, the brain relies on an extensive network of blood vessels, with efficient communication between the brain parenchyma and circulatory system, adjusting local blood flow to energetic demand according to the activity in different brain regions (Fouda et al., 2019).

**FIGURE 2:**
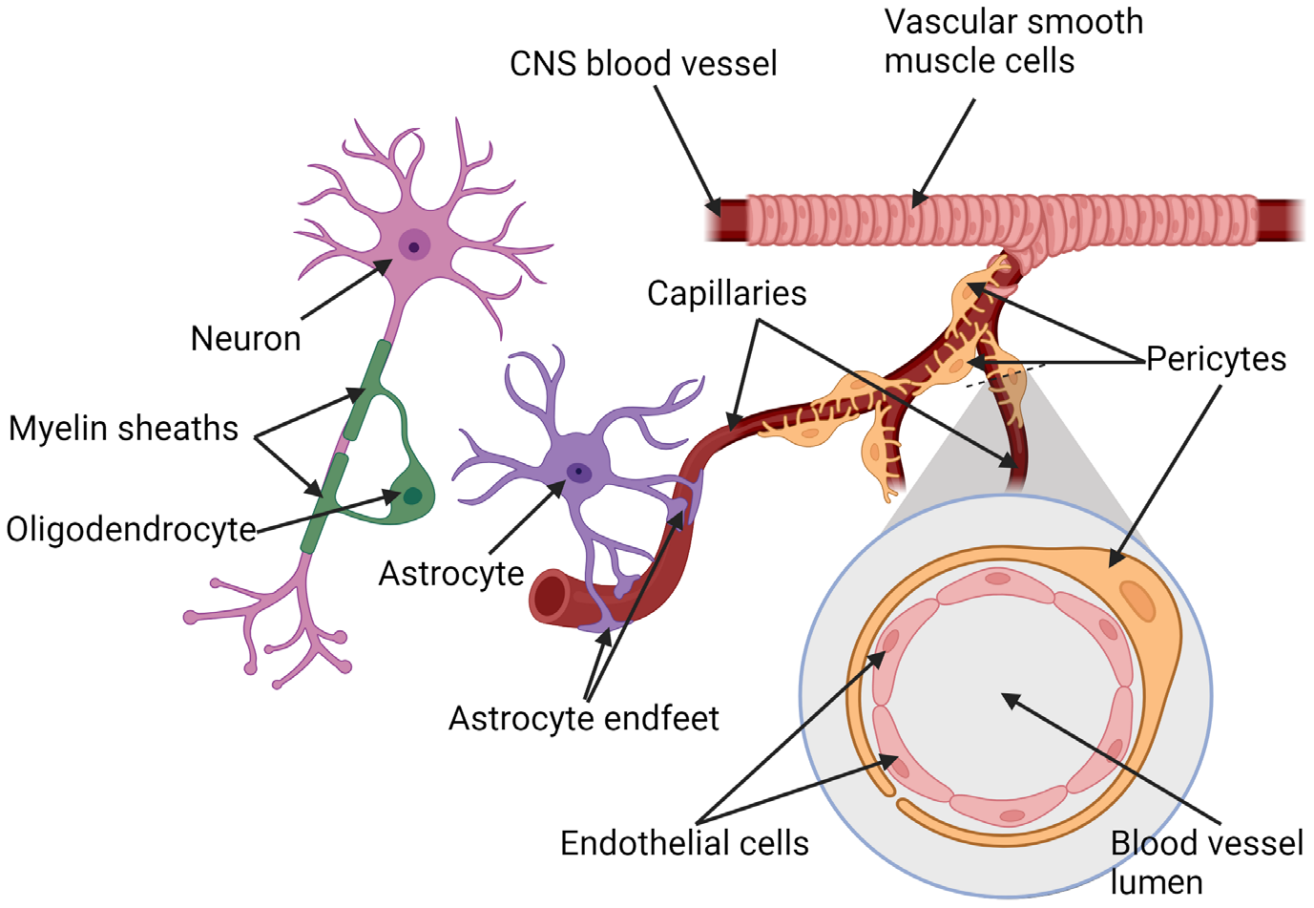
Components of the neurovascular unit. Central nervous system (CNS) blood vessels are surrounded by contractile vascular smooth muscle cells. Substance exchange occurs across the capillaries which are surrounded by pericytes. Astrocyte endfeet embrace the capillaries, forming a key element of the blood–brain barrier that regulates substance exchange with the blood. Astrocytes also reach out to oligodendrocytes and neurons, passing along trophic support. Oligodendrocytes form myelin sheaths and provide metabolic support to neurons. Created with BioRender.

Regulation of blood flow is executed at the level of the microvasculature by the neurovascular unit (NVU), which is composed of endothelial cells (EC), vascular smooth muscle cells, pericytes, and astrocytes (Figure 2) (Schaeffer & Iadecola, 2021), to insure the proper supply of nutrients to oligodendrocytes and neurons. Active communication, known as neurovascular coupling (NVC), between the NVU and CNS parenchymal cells coordinates regional blood flow (Kaplan et al., 2020).

### 4.1 Vascular smooth muscle cells

Vascular smooth muscle cells are contractile and can alter blood vessel diameter, providing a regulatory mechanism to modulate cerebral blood flow. Changes in oxygen and nutrient levels, sensed by oligodendrocytes and neurons through signaling pathways regulated by AMPK, TGF‐β, and HIF trigger a signaling cascade that induces vascular smooth muscle cell actomyosin‐dependent contraction by modulating intracellular Ca^2+^ (Hayes et al., 2022; Wilson & Matschinsky, 2020). Vascular smooth muscle cells also respond to mechanical forces and vascular pressure (Liu & Lin, 2022; Na et al., 2008).

### 4.2 Pericytes

Brain capillaries, however, are not surrounded by vascular smooth muscle cells, but instead, pericytes extend specialized arms that wrap the capillary epithelium (see Figure 2). Pericytes are also capable of contraction and regulate blood flow and the flux of nutrients and other signaling molecules between oligodendrocytes, other CNS cells, and the circulation (Alarcon‐Martinez et al., 2020; Armulik et al., 2011). Neuronal activity and glutamate elicit pericyte relaxation, increasing local blood flow (Hall et al., 2014). Pericytes are lost during aging, contributing to vascular‐mediated neurodegeneration and disruption of white matter, causing loss of myelin and oligodendrocytes (Bell et al., 2010; Montagne et al., 2018). Pericyte dysfunction and loss are also observed in the early stages of Alzheimer's disease (AD) (Apátiga‐Pérez et al., 2022; Nortley et al., 2019; Shi et al., 2020). Pericytes may also contribute to neurodegeneration through excessive capillary constriction, modulating immune reactivity, and by participating in glial scar formation (Cheng et al., 2018).

### 4.3 Astrocytes

Central nervous system (CNS) vascular epithelial cells are surrounded by astrocyte endfeet that form an interface between the circulating blood with brain parenchymal cells that regulate the interchange of nutrients, signaling molecules, and other substances (Abbott et al., 2006). Astrocytes influence the BBB (Davson & Oldendorf, 1967), regulating tight junctions and the expression of transporter molecules and enzymes (Dehouck et al., 1990; Haseloff et al., 2005; Hayashi et al., 1997; McAllister et al., 2001; Rubin et al., 1991; Schinkel, 1999; Sobue et al., 1999). Neurons consume much more energy than glial cells to execute their functions (Li & Sheng, 2022). To support this demand, like oligodendrocytes, astrocytes share a portion of their energetic resources with neurons. This is accomplished by oxidative glycolysis, a relatively low‐efficiency mechanism for producing ATP. Lactate, an end product of glycolysis, is then shuttled to neurons where it is used for mitochondrial respiration, a substantially more productive mechanism for ATP synthesis (Bélanger et al., 2011; Bonvento & Bolaños, 2021; Dienel, 2019). Astrocytes also store glycogen, providing a bank of stored capacity that can be accessed when needed (Alberini et al., 2018; Brown & Ransom, 2015; Falkowska et al., 2015). Astrocytes and oligodendrocytes are connected metabolically and share energetic substrates via gap junctions composed of connexin47 and connexin30 expressed by astrocytes, and connexin47 and connexin32 expressed by oligodendrocytes (Tress et al., 2012). This pan‐glial network also provides critical support for oligodendrocyte development during myelination (Nave, 2010).

Taking into account the energetic requirements of the CNS, the perfect functioning of the NVU is essential for brain health, and impairment of this system can have serious detrimental consequences. In this regard, particular attention should be given to possible dysregulation of the NVU, which may contribute to metabolic stresses that underlie the etiology of demyelinating disease.

## 5 IMPACT OF METABOLIC STRESS ON NVU

Blood flow is dynamically regulated in different regions of the CNS by the NVU in response to local energy demand. From the point of view of a myelinating oligodendrocyte, executing this regulation depends on communication with other brain parenchymal cells and those that compose the brain vasculature.

Many studies have highlighted the important role of astrocytes in regulating the blood–brain barrier. To increase blood supply in ischemic conditions, astrocytes down‐regulate or disrupt endothelial cell tight junction proteins by secreting matrix metalloproteinase‐9 (MMP9), vascular endothelial growth factor A (VEGF‐A), chemokine C‐X‐C motif ligand 1 (CCL1), chemokine monocyte chemoattractant protein‐1 (MCP1), and the peptide glutamyl‐cysteinyl‐glycine (GSH) (Gao et al., 2023; Huang et al., 2020). Astrocytes may also stimulate expression of Delta‐like Notch ligand DLL4 by endothelial cells, which in turn increases expression of glutamate transporter 1 (GLT1) and glutamate/aspartate transporter (GLAST) by astrocytes, promoting the interaction of both cell types and strengthening the blood–brain barrier (Martinez‐Lozada & Robinson, 2020). In contrast, inhibiting Wnt signaling in astrocytes weakens the blood–brain barrier by disrupting astrocytic endfeet (Gao et al., 2023; Guérit et al., 2021), interrupting the nutrient supply with deleterious consequences for oligodendrocytes.

Increased neural activity results in elevated energetic demand, with hypoglycemia indicating that supply is insufficient. These two phenomena engage mechanisms to promote vasodilation. In contrast, when the body is mobilized for strenuous physical activity and the sympathetic nervous system is activated, angiotensin II induces CNS vasoconstriction. Ca^2+^ and K^+^‐dependent signaling pathways in blood–brain barrier astrocytes modulate this vasodilation and vasoconstriction (Boily et al., 2021; Butt & Kalsi, 2006; Gao et al., 2023; Knot & Nelson, 1998; Nippert et al., 2022).

Further studies are required to fully elucidate how oligodendrocytes and the NVU communicate and how this may be disrupted to cause metabolic stress.

## 6 ENERGY SOURCES USED BY OLIGODENDROCYTES

### 6.1 Glucose

**FIGURE 3:**
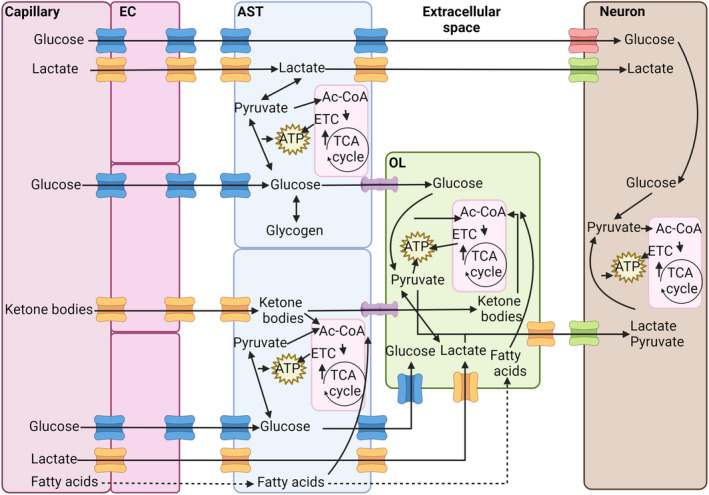
Flux of energetic metabolites in the central nervous system (CNS). Glucose, lactate, ketone bodies, and fatty acids pass into the CNS via endothelial cells and are then transferred to astrocytes, oligodendrocytes, and neurons. Glucose trafficking is mediated by glucose transporter (GLUT) 1 (blue channels) in endothelial cells, astrocytes, and oligodendrocytes and by GLUT3 (red channels) in neurons. Lactate and ketone bodies pass through MCT1 in endothelial cells, astrocytes, and oligodendrocytes (orange channels) and MCT4 in neurons. Fatty acids can diffuse directly through plasma membranes. Metabolites can be shared between astrocytes and oligodendrocytes through gap junctions formed by connexins (purple). Glucose can be converted and stored in astrocytes as glycogen. Glucose and lactate can be converted into pyruvate by glycolysis to yield ATP. Pyruvate, ketone bodies, and fatty acids can be converted into acetyl‐CoA (Ac‐CoA) and used to produce considerable amounts of ATP by oxidative phosphorylation in mitochondria via the TCA cycle and the electron transport chain (ETC). This source of ATP is particularly important for neurons because of their higher energetic demands. Fatty acids are mainly used by oligodendrocytes to produce myelin, as is a substantial portion of the acetyl‐CoA produced from other metabolites. Created with BioRender.

**FIGURE 4:**
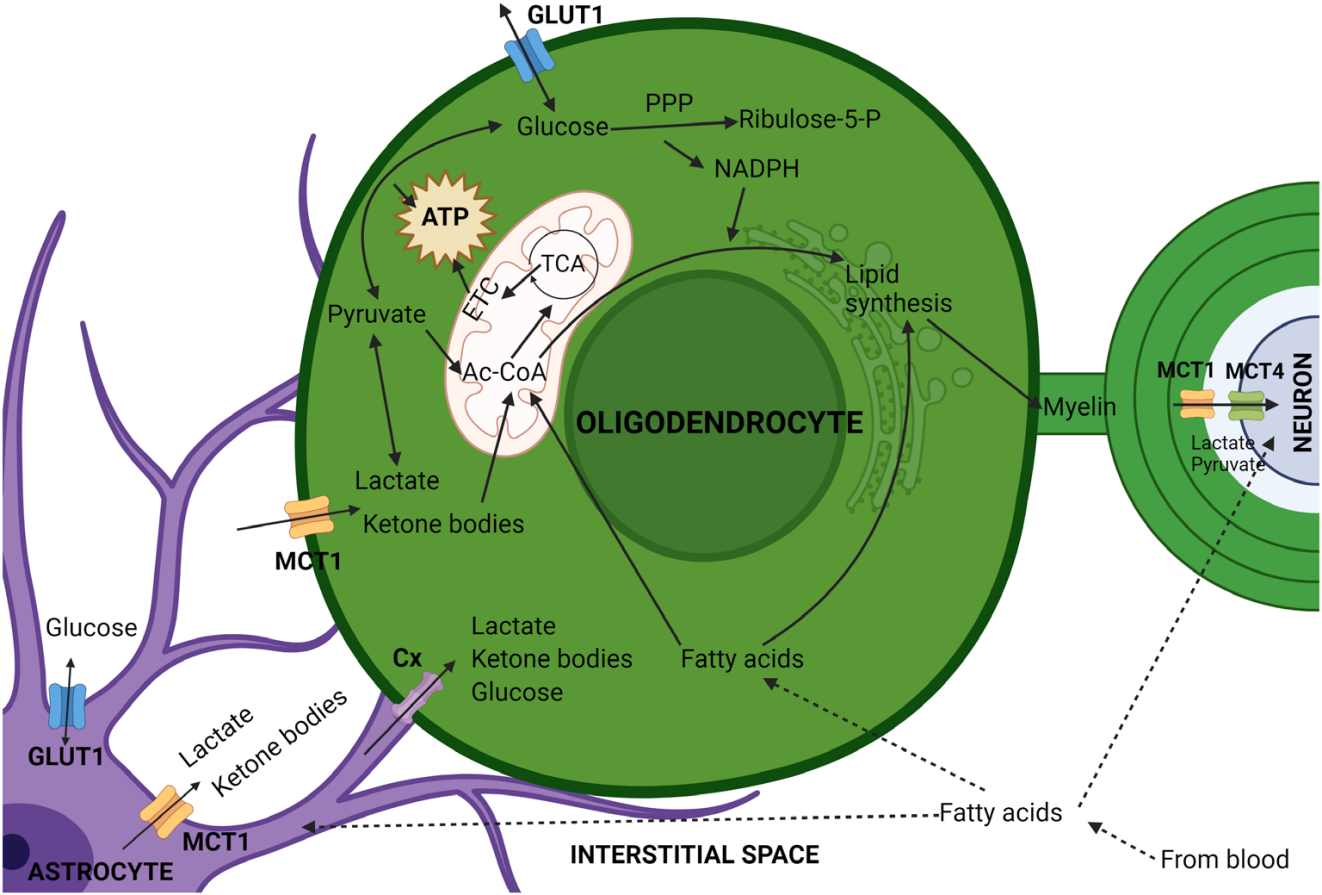
Oligodendrocyte energy metabolism and its relationship with neighboring cells. Oligodendrocytes import glucose from the central nervous system interstitial space via glucose transporter (GLUT) 1, and lactate and ketone bodies by monocarboxylate transporter (MCT) 1. These metabolites are transferred to the interstitial space by astrocytes largely using the same channels, or they can be transferred to oligodendrocytes via gap junctions formed by connexins (Cx). Once taken up, glucose can be converted to pyruvate through glycolysis, generating ATP, or feed the pentose phosphate pathway (PPP), generating NADPH, an important metabolite for lipid synthesis and the cellular anti‐oxidant system. Lactate can also be converted to pyruvate, which in turn can be converted to acetyl‐CoA (Ac‐CoA) in mitochondria. Ketone bodies may also be converted to Ac‐CoA, which can be used to produce ATP by oxidative phosphorylation involving the TCA cycle and electron transport chain (ETC). Alternatively, in oligodendrocytes a substantial portion of cellular Ac‐CoA is used for lipid synthesis to produce myelin. Fatty acids can diffuse through the cell plasma membrane and generate Ac‐CoA by β‐oxidation, or be used for lipid synthesis. Lactate, shuttled to the periaxonal space by oligodendrocytes can be taken up by neurons to support their energetic needs.

Glucose is the best‐understood energy metabolite in the CNS and an essential nutrient for oligodendrocyte survival and function (Mergenthaler et al., 2013). Cellular uptake of glucose occurs through specific glucose transporters (GLUT) (Koepsell, 2020). To enter the brain, glucose must cross the blood–brain barrier, passing capillary epithelial cells that are surrounded by astrocyte endfeet (Koepsell, 2020; Morgello et al., 1995). GLUT1 is the main GLUT expressed by endothelial cells, distributed along luminal and abluminal sides, and by oligodendrocytes and astrocytes (Koepsell, 2020; Morgello et al., 1995). Glucose is released from epithelial cells and astrocytes by GLUT1 into brain parenchymal extracellular space (Cornford & Hyman, 2005; Koepsell, 2020; Leino et al., 1997). Uptake into neurons occurs mainly via GLUT3 (Koepsell, 2020; Maher et al., 1996). Oligodendrocytes also take up glucose from the extracellular space but predominantly express GLUT1 (Jha & Morrison, 2018; Maher, 1995). GLUT3 has a higher affinity for glucose compared to GLUT1, suggesting that GLUT1 is a rate‐limiting component in the rate of glucose transport (Figures 3 and 4) (Jha & Morrison, 2018).

GLUT1 and GLUT3 are regulated transcriptionally and post‐transcriptionally, primarily in response to glucose and oxygen levels (Jha & Morrison, 2018). GLUT1 expression also responds to growth factors present in serum, in particular platelet‐derived growth factor (PDGF), fibroblast growth factor (FGF), epidermal growth factor (EGF), insulin, insulin‐like growth factor 1 (IGF‐1), and transforming growth factor‐β (TFG‐β) (McGowan et al., 1995). Glutamate regulates GLUT1 and GLUT3 expression in different directions, increasing GLUT1 in astrocytes, while decreasing GLUT3 in neurons (Jha & Morrison, 2018). Notably, GLUT1 expression in response to insulin is not equal in all cell types (McGowan et al., 1995) and is differently influenced by other hormones. Growth hormone (GH) inhibits GLUT1 expression, while triiodothyronine (T_3_) has the opposite effect (McGowan et al., 1995). GLUT2 and GLUT4 are up‐regulated in some brain regions in response to glucose and insulin (Jha & Morrison, 2018). Post‐transcriptionally, the stability of GLUT1 mRNA is modulated by several factors, including TNF and arachidonic acid (McGowan et al., 1995). Oligodendrocytes express N‐methyl‐D‐aspartate (NMDA) receptors and are sensitive to axonal glutamate release, which promotes translocation of GLUT1 to the oligodendrocyte plasma membrane (Saab et al., 2016).

Once taken up, there are important differences in how glucose is utilized by oligodendrocytes, astrocytes, and neurons (Figure 4). Astrocytes process a relatively large amount of glucose via glycolysis and the TCA cycle compared to oligodendrocytes and neurons. Oligodendrocytes direct a considerable proportion of the glucose they consume to the pentose phosphate pathway (PPP) (Sánchez‐Abarca et al., 2001), a metabolic pathway composed of oxidative and non‐oxidative branches that is used by cells to produce precursors for nucleotide and amino acid synthesis. The oxidative branch is unidirectional, using glucose‐6‐phosphate as the initial substrate to yield ribulose‐5‐phosphate and NADPH, while the non‐oxidative branch produces ribose 5‐phosphate and sugar phosphates for amino acid synthesis (Stincone et al., 2015). NADPH, derived from the PPP, is required for the synthesis of thioredoxin/peroxiredoxin and glutathione, critical cellular anti‐oxidants. Insufficient levels of NADPH increase cell vulnerability to oxidative stress (Stincone et al., 2015). PPP inhibition with 6‐amino‐NADP (6AN) causes OPC and oligodendrocyte death because of depletion of NADPH and glutathione. 6AN toxicity can be attenuated by treatment with the anti‐oxidant trolox (Kilanczyk et al., 2016), supporting the conclusion that cell death results from oxidative stress. NADPH is also important for the synthesis of cholesterol and fatty acids (Chandel, 2021) and therefore essential for the production of myelin. A recent study reports that lactate dehydrogenase expression is down‐regulated in mature oligodendrocytes. This reduces the conversion of pyruvate to lactate and increases the capacity of oligodendrocytes to shuttle pyruvate to neurons, along with lactate transferred from astrocytes (Späte et al., 2024). Additional studies are needed to fully appreciate the implications of this to oligodendrocyte metabolism and axonal function.

### 6.2 Lactate

In addition to glucose, oligodendrocytes can take up and use other metabolites to produce ATP. Lactate, a three‐carbon metabolite, is interchangeably converted into pyruvate by lactate dehydrogenase (LDH) with conversion of NAD^+^ into NADH in parallel (Rabinowitz & Enerbäck, 2020). Pyruvate can further be converted into acetyl‐CoA, which enters the TCA cycle to produce ATP in mitochondria (Shi & Tu, 2015). Acetyl‐CoA can also be directed toward lipid synthesis (Shi & Tu, 2015). Oligodendrocytes, as well as astrocytes and neurons, are capable of lactate uptake, using it to produce ATP and lipids (Sánchez‐Abarca et al., 2001). Compared to neurons and astrocytes, oligodendrocytes have a higher capacity for lactate uptake, which is used mainly for lipogenesis (Shi & Tu, 2015).

Lactate and pyruvate, ketone bodies, and other monocarboxylates (molecules that have one carboxylate group) are taken up by cells via monocarboxylate transporters (MCTs). There are 14 MCTs, of which MCT1‐4 are the best characterized (Felmlee et al., 2020). Glial cells express mainly MCT1 and MCT4, while neurons express MCT2 (Figures 3 and 4). Higher mRNA expression was found in oligodendrocytes compared to astrocytes, but it is not clear if this translates to higher levels of protein (Lee et al., 2012).

MCT1 is also expressed by brain endothelial cells, supporting the exchange of lactate and other monocarboxylates with the circulating blood (Figure 3). In endothelial cells, MCT1 is regulated by PTEN/AKT signaling, and disrupting this pathway causes lactate accumulation in the brain, with multiple effects, including impairment of hippocampal neurogenesis, as lactate inhibits the differentiation of neural stem cells (Wang et al., 2019).

Oligodendrocyte metabolism switches during development, importing glucose and lactate during myelination to support lipid production, but subsequently becoming predominantly glycolytic after maturation (Fünfschilling et al., 2012; Rinholm et al., 2011). Because of this glycolytic‐bias, mature myelinating oligodendrocytes release glycolytic products into the periaxonal space via MCT1, transferring them to support neuronal activity (Lee et al., 2012). Neurons are highly dependent on lactate for their metabolism, as they direct glucose to the PPP to support the generation of anti‐oxidants (Barros, 2013). Oligodendrocytes detect elevated extracellular K^+^ that results from axonal activity. This induces oligodendroglial Ca^2+^ uptake, which stimulates glycolysis, supporting the supply of glycolytic products to neurons (Looser et al., 2024). Sirtuin 2, a deacetylase that promotes mitochondrial function, is transferred via exosomes from oligodendrocytes to axons, increasing axonal ATP (Chamberlain et al., 2021). MCT1 inhibition or knock‐down can produce hypomyelination, increase neuronal death, and cause axonopathy, illustrating the dependence of neurons on glycolytic products supplied by oligodendrocytes (Lee et al., 2012; Philips et al., 2021). Reduced axonal ATP has also been observed with defects in myelin in a mouse with spastic paraplegia (Trevisiol et al., 2020).

Oligodendrocytes also consume lactate for their own energetic demands (Sánchez‐Abarca et al., 2001). Lactate supports myelination in low glucose conditions (Rinholm et al., 2011) and enhances proliferation and differentiation of OPCs (Ichihara et al., 2017). MCT1 has a key role supporting the response of OPCs and oligodendrocytes to metabolic stress, such as oxygen–glucose deprivation caused by cerebral ischemia, supporting the conclusion that lactate is protective under such conditions (Zhou et al., 2018).

Studies that have largely focused on neurons indicate that lactate can also serve as a signaling molecule. Lactate levels in the tissue surrounding a neuron vary dynamically according to the neuron's activity. Glutamate and K^+^ release stimulate GLUT1 expression and glycolysis in nearby astrocytes. Lactate diffuses more broadly, inhibiting the activity of nearby neurons and promoting hyperemia to redirect glucose and oxygen to active neurons (Barros, 2013). Lactate signaling also impacts the regulation of cerebral blood flow (Gordon et al., 2008; Rinholm & Bergersen, 2014). In neurons, lactate influences the activity of NMDA receptors by increasing cytosolic NADH, regulating gene expression associated with memory formation and retention (Vaccari‐Cardoso et al., 2022). Lactate also influences neuronal activity via G‐protein coupled receptors, in particular hydroxycarboxylic acid receptor 1 (HCA1, previously known as G protein‐coupled receptor 81(GPR81)), although it is not clear if this signaling exerts an inhibitory or excitatory effect (Vaccari‐Cardoso et al., 2022). Recent studies suggest that HCA1 has neuroprotective and immunomodulatory roles (Colucci et al., 2023; Luo et al., 2022). HCA1 and NMDA receptors are also expressed by oligodendrocytes (Rinholm & Bergersen, 2014), although the function and regulatory effect of lactate via these receptors require further investigation. It was recently demonstrated that lactate can influence gene expression by being covalently linked to lysine in histones, a process called histone lactylation (Zhang et al., 2019). This post‐translational modification is putatively involved in hypoxic–ischemic inflammation and macrophage activation (Zhang et al., 2019). Lactate also activates HIF‐1α in dendritic cells, limiting CNS autoimmunity (Sanmarco et al., 2023).

Because of the relatively high energy content of lactate, it will likely be informative in future studies to investigate how oligodendrocytes and other CNS cells may utilize this metabolite either for energetic needs or in signaling, particularly concerning interactions with the immune system.

### 6.3 Fatty acids

Fatty acids can be taken up from the blood by endothelial cells and passed through the BBB by astrocytes to oligodendrocytes. While fatty acids can diffuse through the plasma membrane, cellular adsorption can be enhanced by fatty acid translocase (CD36), fatty acid transport proteins (FATP), and fatty acid binding proteins (FABPs). Both oligodendrocytes and OPCs express FATP4, while OPCs also express FABP7 and oligodendrocytes express FABP5. Ablation of these proteins impairs OPC proliferation and oligodendrocyte differentiation (Poitelon et al., 2020). Fatty acids derived from the circulation can support myelination, although they cannot fully substitute for specialized lipids that are internally synthetized by oligodendrocytes (Dimas et al., 2019). Interestingly, oligodendrocyte myelination also relies on lipids synthesized and supplied by astrocytes, and when this synthesis is impaired, fatty acids derived by oligodendrocytes from the circulation may compensate (Figures 3 and 4) (Camargo et al., 2017).

Fatty acids can be converted to acetyl‐CoA by β‐oxidation and have a particularly high energy content, with the potential to generate up to 129 molecules of ATP from a 16‐carbon fatty acid (Bhagavan, 2002). However, because of the high demand for lipids to produce and maintain myelin, brain metabolism is biased away from using fatty acids to generate energy. Additionally, fatty acid β‐oxidation requires more oxygen than metabolizing glucose and generates ROS as toxic by‐products. A further disadvantage of this mechanism as a source of energy is that ATP generation is considerably slower compared to glycolysis (Schönfeld & Reiser, 2013). In spite of these limitations, fatty acid oxidation is detected in brain, particularly by astrocytes (Ebert et al., 2003). Studies in Schwann cells, the myelinating glia of the peripheral nervous system, show that high glucose increases fatty acid oxidation (Smit, 2019). Fatty acids are stored as lipid droplets (LD), mainly in astrocytes, but also in oligodendrocytes during development; however, these stored lipids are mainly used for myelin production (Smolič et al., 2021). LDs can accumulate in glial cells, particularly during aging, in response to nutrient deprivation, hypoxia, excess of fatty acids or lactate, and this accumulation is linked to neurodegenerative disorders (Smolič et al., 2021). The extent to which fatty acids may be utilized as a source of energy by oligodendrocytes remains unclear.

Myelin is a lipid‐rich material and has an energy content that could potentially be used as a reservoir of stored energy. It has been proposed that this material can be used by oligodendrocytes during metabolic stress, helping to explain their resistance to cell death under these conditions, and also mobilized to transfer glycolytic products to neurons, supporting their energetic needs under deprived conditions (Asadollahi et al., 2022). Intriguingly, looking from an evolutionary perspective, lampreys are primitive vertebrates that do not possess oligodendrocytes or myelin, yet axons in the lamprey CNS are ensheathed by glial processes that contain large amounts of lipid droplets, which have been speculated to be an evolutionary precursor of myelin (Weil et al., 2018). It will be interesting to investigate this hypothesis further and determine to what extent myelin and lipid droplets may share similar biochemical characteristics, particularly in relation to their capacity to store energy.

### 6.4 Ketone bodies

Ketone bodies are monocarboxylates that possess ketone groups. These include acetoacetic acid and β‐hydroxybutyrate and are produced from fatty acids, mainly in the liver, but also by astrocytes (Silva et al., 2022; Cahill Jr. & Veech, 2003). Like lactate, ketone bodies can also be transported into cells by MCT1. Ketone bodies produced in astrocytes can be exported by MCT1 and MCT4 to the extracellular space and then taken up by oligodendrocytes via MCT1 and by neurons using MCT2 (Pierre & Pellerin, 2005). Conversion of ketone bodies into acetyl‐CoA allows them to fuel the production of ATP by oxidative phosphorylation or feed lipid synthesis for myelin (Figures 3 and 4) (Tepavčević, 2021).

Ketone bodies are important alternative energy substrates, particularly during fasting (Pierre & Pellerin, 2005). They are also an important nutrient during development, as detected levels of ketone bodies in blood were up to 10 times higher in developing rats compared to adults, likely as a result of the relatively high energetic demand, as the developing brain consumes three times more energy compared to an adult brain (Tepavčević, 2021). Oligodendrocytes are the main consumers of ketone bodies in the brain, utilizing them for oxidative phosphorylation and for the synthesis of lipids and cholesterol (Sykes et al., 1986).

A ketogenic diet can increase the amount of ketone bodies circulating in blood. Several studies highlight that a ketogenic diet can be beneficial for brain health, being protective against axonal injury, cognitive impairment, neuroinflammation, and tissue loss following traumatic brain injury (Mu et al., 2022; Thau‐Zuchman et al., 2021; Wang et al., 2023). Other studies provide evidence that a ketogenic diet may have benefits for demyelinating diseases such as MS and Pelizaeus‐Merzbacher disease (Stumpf et al., 2019; Sun et al., 2022).

## 7 OTHER METABOLITES INVOLVED IN OLIGODENDROCYTE ENERGY SUPPLY

### 7.1 Glycogen

Glycogen is a polymer composed of glucose subunits that is used by cells to store energy (Roach et al., 2012). In humans, it is stored mainly in muscle and liver but is also present in the brain (Jensen et al., 2011). In the CNS, glycogen is synthesized and stored almost exclusively in astrocytes (Brown et al., 2019). In astrocytes, glycogen is cleaved into glucose‐1‐phosphate by glycogen phosphorylase and then transformed to ATP and pyruvate by glycolysis (Roach et al., 2012). Pyruvate is then converted to lactate and shuttled to neurons (Figures 3 and 4) (Bonvento & Bolaños, 2021). Astrocytic glycogen allows the CNS to cope with short‐term mismatches in the supply and demand of energy, such as reduced local availability of glucose and lactate because of an increase in neural activity (Brown et al., 2019; Brown & Ransom, 2015). In this way, astrocytes support the availability of lactate in the periaxonal space by storing glycogen and mobilizing glucose when needed. While this may primarily support neuronal activity, it likely also contributes to oligodendrocytes, although the significance of astrocytic stores of glycogen to the energetic needs of oligodendrocytes remains relatively poorly understood.

Glycogen synthase (GS) is the rate‐limiting enzyme in the glycogen synthesis pathway. Its expression is regulated by vasoactive intestinal peptide, noradrenaline, and adenosine. GS is also activated by insulin and glucose‐6‐phosphate, linking the availability and uptake of glucose by astrocytes to glycogen accumulation (Pederson, 2019). In contrast, glycogen degradation is controlled by glycogen phosphorylase (GP), which is activated by glucagon and noradrenaline. GP is also activated by a high AMP/ATP ratio, indicating low cellular energy. High levels of glucose‐6‐phosphate, consistent with high levels of glycolysis, inhibit its activity (Mathieu et al., 2019).

It is still a matter of debate if oligodendrocytes can produce and store glycogen and if so, in which conditions this may occur.

### 7.2 Taurine and creatine

Taurine is an amino acid that supports oxidative phosphorylation by stabilizing mitochondrial pH. Taurine promotes oligodendrocyte differentiation and increases the availability of serine, a crucial amino acid for the synthesis of myelin components. It also protects the cell against apoptosis induced by metabolic stress by increasing the ratio of BCL2 to BAX protein expression (Rosko et al., 2019; Zhang et al., 2016). Although not directly used as a substrate for ATP production, taurine supports oligodendrocyte energy generation and myelination (Hansen et al., 2010; Rosko et al., 2019; Xu et al., 2015).

Creatine is a metabolite with the capacity to store a phosphate group for transfer to ADP to generate ATP, serving as a useful energy source for short periods of intense activity (Bonilla et al., 2021). Creatine cannot cross the BBB and must be synthesized within the CNS. This is performed by L‐arginine: glycine amidinotransferase (AGAT), which is expressed by all glial cells, and guanidinoacetate methyltransferase (GAMT), which is mainly expressed by oligodendrocytes. Once synthesized by glia, creatine can be transported to neurons by SLC6A8, which is expressed by neurons and oligodendrocytes (Braissant et al., 2001, 2010; Rosko et al., 2019).

## 8 PATHOLOGICAL IMPLICATIONS OF FAILURE TO CONTROL THE SUPPLY OF NUTRIENTS AND GROWTH FACTORS IN THE CNS

### 8.1 NVU disruption and white matter disease

#### 8.1.1 Acquired

Pathological processes affecting the brains small vessels and capillaries are grouped together under the term cerebral small vessel disease. Damage from these processes includes white matter lesions among other deteriorations of brain structure, contributing to cognitive decline in older individuals (Pantoni, 2010). Ischemic‐like conditions are thought to be the main pathological feature of small vessel diseases (van der Knaap & Bugiani, 2017), with chronic white matter hypoperfusion resulting from restriction of blood flow in small vessels, producing oligodendrocyte injury and myelin degeneration (Pantoni, 2002, 2010; Petito et al., 1998).

#### 8.1.2 Genetic

Leukodystrophies are genetic disorders of CNS white matter that often take a progressive course. Although these diseases are largely characterized by demyelination, they are not always related to the mutation of a gene that is directly oligodendrocyte‐associated. Disruption of NVU components that are essential for oligodendrocyte support, as described, may contribute to the origin of a leukodystrophy (van der Knaap & Bugiani, 2017). In particular, leukodystrophies of vascular origin involve the disruption of the proper supply of nutrients to the CNS, contributing to myelin degeneration and oligodendrocyte death. Cerebral autosomal dominant arteriopathy with subcortical infarcts and leukoencephalopathy (CADASIL), cerebral autosomal recessive arteriopathy with subcortical infarcts and leukoencephalopathy (CARASIL), and hereditary cerebral amyloid angiopathy are rare genetic diseases associated with cerebral small vessel disease and white matter deterioration (van der Knaap & Bugiani, 2017). Cathepsin A‐related arteriopathy with strokes and leukoencephalopathy (CARASAL) is a cerebral small vessel disease associated with a recessive mutation in the gene encoding cathepsin A that causes galactosialidosis, a lysosomal storage disorder (Caciotti et al., 2013; van der Knaap & Bugiani, 2017).

Leukoencephalopathy with vanishing white matter disease is a recessive genetic disorder characterized by progressive deterioration of CNS myelin that may be caused by mutation of eukaryotic initiation factor 2B (eIF2B) (van der Knaap et al., 2006). How this eIF2B mutation results in the characteristics of this disease is not clearly understood but has been attributed to a primary impact on astrocytes and is considered an example of astrocytopathy. The severe white matter deterioration occurs in parallel with abnormal changes in astrocyte morphology (Bugiani et al., 2018). This suggests a deficiency in normal astrocytic functions or acquisition of a pathologic phenotype. The genetic defect can result in the activation of the unfolded protein response with constitutive predisposition to cellular stress. In addition, changes in the expression of components of the respiratory chain have been identified which could impair mitochondrial performance and ATP production (Bugiani et al., 2018; Raini et al., 2017).

The X‐linked form of Charcot–Marie–Tooth disease is a peripheral neuropathy that manifests only in males and has symptoms that include muscle weakness and atrophy, reflex loss, and sensory abnormalities (Scherer & Kleopa, 2012). Patients with this disease because of mutation of the GJB1 gene, which encodes connexin32, may present with encephalopathy and changes in CNS myelin (Scherer & Kleopa, 2012). Loss of function deletions of GJB1 are proposed to cause demyelination (Gonzaga‐Jauregui et al., 2010). Mutations that reduce connexin 32 expression disrupt gap junctions between astrocytes and oligodendrocytes, and when combined with neuroinflammation, induce ER stress in oligodendrocytes (Olympiou et al., 2016). Gap junctions formed by connexins underlie the pan‐glial network in the brain parenchyma and are critically involved in the exchange of energetic metabolites (Saab et al., 2013), with genetic defects in connexins linked to failure of energetic metabolism.

### 8.2 Hypoperfusion, metabolic stress, and multiple sclerosis: Does hypoperfusion contribute to demyelination in MS?

Immune‐related aspects of MS are well characterized and all current treatments target the autoimmune component of the disease. These treatments have proven effective against relapsing–remitting multiple sclerosis; however, progressive MS continues to resist current systemic immune therapies (Doshi & Chataway, 2016). Substantial evidence supports the hypothesis that the course of MS involves more than a systemic immune system–mediated mechanism. Histologic studies of MS lesions and surrounding normal‐appearing white matter (NAWM) indicate evidence of ongoing metabolic stress, such as the presence of stress granules in oligodendrocytes and astrocytes, as well as in neurons (D'Haeseleer et al., 2015).

### 8.3 Hypoperfusion in MS

Many studies have provided evidence of reduced perfusion in the CNS associated with MS (Adhya et al., 2006; Brooks et al., 1984; Law et al., 2004; Lycke et al., 1993; Sun et al., 1998; Swank et al., 1983; Varga et al., 2009). Others indicate that hypoperfusion may occur early in MS (Law et al., 2004; Papadaki et al., 2012; Varga et al., 2009), before inflammation, BBB leakage, and plaque formation (Wuerfel et al., 2004). Hypoperfusion is thought to result primarily from vascular impairment and not as a consequence of axonal degeneration induced by reduced metabolic demand (De Keyser et al., 2008; Mascali et al., 2023).

MS lesions preferentially form in brain regions with relatively low blood perfusion (Narayana et al., 2014). A particularly susceptible area is the periventricular white matter (Martinez Sosa & Smith, 2017), which contains long, thin arterioles and is relatively poorly vascularized. Notably, these regions contain end‐arterioles that supply exclusive fields, meaning that the target regions lack a compensatory supply (Martinez Sosa & Smith, 2017). Further, long narrow arteries tend to lose more oxygen through their walls, and this loss is exacerbated in tissues that are hypoxic, which is observed in MS (Martinez Sosa & Smith, 2017).

Hypoperfusion is also seen in the animal model experimental autoimmune encephalomyelitis (EAE), with hypoxia detected in conjunction with neurological deficits and demyelination (Davies et al., 2013). Treatment with nimodipine, a CNS‐specific vasodilator, restored oxygen levels in lesion areas of EAE‐affected mice, with a reduction in demyelination (Desai et al., 2020).

Hypoxia‐inducible factor 1 (HIF1), another indicator of hypoperfusion, is a transcription factor activated in response to hypoxia and ischemia. HIF1 is composed of two subunits (HIF1α and HIF1β). While HIF1β is constitutively expressed, HIF1α expression is regulated by oxygen availability. In normal conditions, HIF1α is rapidly degraded by hydroxylation. In hypoxic conditions, HIF1α accumulates, translocates to the nucleus, and forms a complex with HIF1β to increase the expression of target genes that promote cell survival (Benarroch, 2009; Correia & Moreira, 2010). Inflammatory responses and vascular permeability can lead to increased HIF1α expression (Peyssonnaux et al., 2007; Thiel et al., 2007; Weidemann et al., 2009). In MS, HIF1α up‐regulation has been detected in white matter pre‐demyelinating lesions (Graumann et al., 2003; Zeis et al., 2008). Further, high HIF1α expression was detected in MS lesions with oligodendrocytes presenting dying‐back oligodendropathy (Lassmann, 2003). These findings provide evidence that up‐regulation of HIF1 is an early indication of developing hypoperfusion in MS.

Axon demyelination can also exacerbate oligodendrocyte injury because of enhanced hypoxia. Demyelinated axons require more energy to maintain neural transmission, which over activates neuronal mitochondria. It is possible that this abnormally increased consumption of oxygen results in scarcity in the local microenvironment, damaging the neuron itself and also nearby oligodendrocytes (Aboul‐Enein et al., 2003; Trapp & Stys, 2009).

It is critical that future studies address how hypoperfusion may be influenced by internal changes in oligodendrocytes, other CNS cells, or by external systemic factors, including MS‐associated immune activation.

### 8.4 Potential causes of cerebral hypoperfusion in MS

Hypoperfusion in MS likely reflects the interplay between inflammation and components of the NVU. It is likely that hypoxia exacerbates inflammation and vice versa, creating a “hypoxia– inflammation cycle” that contributes to tissue injury (Chen et al., 2021; Halder & Milner, 2021; Yang & Dunn, 2018).

Previous studies have shown that inflammation, induced for example with LPS, causes oligodendrocyte loss and reduced mitochondrial activity (Fan et al., 2013; Kaizaki et al., 2013; Yeh et al., 2021). In mice, IFN‐γ impairs oligodendroglial energetic metabolism, reducing glycolysis and oxidative phosphorylation (Minchenberg & Massa, 2019). TNF‐α can induce mitochondrial fragmentation, inhibit mitochondrial calcium uptake, and reduce mitochondrial membrane potential (Bonora et al., 2014; Luo et al., 2017). In turn, mitochondrial dysfunction can promote the release of cytokines from oligodendrocytes, potentially altering microglia activation (Scheld et al., 2019). Such findings provide evidence that inflammation may have a negative impact on the capacity of oligodendrocytes to generate energy.

Possible causes of cerebral hypoperfusion in MS include edema and disturbances of microcirculation, leading to BBB breakdown (Engelhardt, 2008; Göbel, Kraft, et al., 2016; Göbel, Pankratz, et al., 2016; Kermode et al., 1990; Lassmann, 2003; Ryu et al., 2018), all of which are detected in MS lesions (Kermode et al., 1990; Kwon & Prineas, 1994). Inflammation of the vessel wall, which may be caused by antibodies reacting against antigens present in the vessel or cytokines released by leukocytes, may also contribute to the hypoxia‐like conditions in MS lesions (Kopp et al., 1997; Lassmann, 2003; Millan et al., 1997; Mosevoll et al., 2018; Olsson et al., 2021). Inflammation can trigger the clotting cascade and induce microvascular thrombosis with impairment of microcirculation (Kopp et al., 1997; Lassmann, 2003; Millan et al., 1997; Olsson et al., 2021).

Appropriate vascular function is critical. It is therefore essential to investigate and identify the underlying causes of hypoperfusion in MS, addressing the impact of the immune system and the communication between oligodendrocytes and the NVU.

### 8.5 Astrocytes and hypoperfusion in the CNS

Disruption of astrocytic function is hypothesized to be a central mechanism that contributes to CNS hypoperfusion (De Keyser et al., 2008). Astrocytes modulate cerebral perfusion by secreting the potent vasoconstrictor endothelin‐1 (ET1) (Nie & Olsson, 1996; Ostrow et al., 2000). Elevated levels of ET1 have been detected in the blood and cerebrospinal fluid of MS patients, in reactive astrocytes in MS plaques, and in the serum of patients recovering from optic neurites (Castellazzi et al., 2019; D'Haeseleer et al., 2015; Haufschild et al., 2001). Patients with MS present an impaired capacity to dilate cerebral arterioles (Marshall et al., 2014; Nie & Olsson, 1996; Ostrow et al., 2000), suggesting that vasoconstriction resulting from ET1 released by reactive astrocytes could contribute to hypoperfusion in MS (D'Haeseleer et al., 2015).

Astrocytes also influence vasodilatation by regulating the concentration of extracellular K^+^ (Butt & Kalsi, 2006; Knot & Nelson, 1998), which may be impaired in MS and contribute to hypoperfusion (De Keyser et al., 2008). Reduced astrocyte expression of β2‐adrenergic receptors has been reported in MS (De Keyser et al., 1999; Zeinstra et al., 2000). Norepinephrine binding to these receptors signals through cAMP to secrete trophic factors and lactate which are taken up by neurons, supporting their energetic metabolism (De Keyser et al., 2004). Deficient β2‐adrenergic receptor expression by astrocytes is predicted to reduce shuttling of lactate to neurons and limit K^+^ release as a result of lower axonal activity and the sensitivity of Ca2^+^‐dependent K^+^ channels to reduced cAMP (Bolton et al., 2006; De Keyser et al., 2004). A reduced concentration of extracellular K^+^ caused by the impairment in this mechanism in the perivascular area could contribute to vasoconstriction and hypoperfusion (De Keyser et al., 2008).

Impaired axonal metabolism has also been suggested to contribute to hypoperfusion in MS. Axon degeneration in MS is related to mitochondria dysfunction and oxidative stress (Cambron et al., 2012). Reduced N‐acetyl aspartate (NAA), indicating reduced axonal metabolism, was detected in NAWM of SPMS patients by quantitative magnetic resonance spectroscopy (Aboul‐Enein et al., 2010). However, no relationship between the reduction of NAA and cerebral blood flow was detected, suggesting that axonal metabolic failure does not directly cause hypoperfusion (Steen et al., 2013). More evidence is needed to determine how impaired axonal metabolism may influence hypoperfusion in MS.

Excitotoxicity triggered by glutamate released by neurons, astrocytes, or macrophages and microglia can also induce ischemic‐like conditions (Lipton, 1998). Altered glutamate homeostasis has been detected in MS lesions and may cause excitotoxic damage to neurons and oligodendrocytes (Werner et al., 2001).

Considering the important role of astrocytes in the supply of energy in the CNS, further study is warranted into how astrocytes impact metabolic function in neurons and oligodendrocytes.

### 8.6 Microglia modulation of nutrient supply

Microglia differentially influence BBB permeability. Pro‐inflammatory factors released by microglia increase BBB permeability, while microglial anti‐inflammatory factors such as IL10 and TGF‐β reduce BBB permeability (Gao et al., 2023; Ronaldson & Davis, 2020). The release of CXCL13/C‐X‐C motif chemokine receptor 5 (CSCR5) by microglia can also inhibit endothelial cell function, and the release of TNFα by microglia can induce endothelial cell necroptosis (Chen et al., 2019; Gao et al., 2023; Zhang et al., 2022). Upon systemic inflammation, microglia protect the BBB by increasing expression of the endothelial cell tight junction protein claudin‐5, but if inflammation is prolonged, microglia phagocytize astrocytic endfeet, resulting in BBB disruption (Haruwaka et al., 2019). Microglia are also involved in angiogenesis by increasing expression of VEGF‐A and VEGF‐B in endothelial cells upon LPS activation and releasing TGFβ1 in extracellular vesicles through the Smad2/3 pathway (Fu et al., 2023; Gao et al., 2023; Zhang et al., 2023).

### 8.7 OPCs promotion of nutrient supply

**FIGURE 5:**
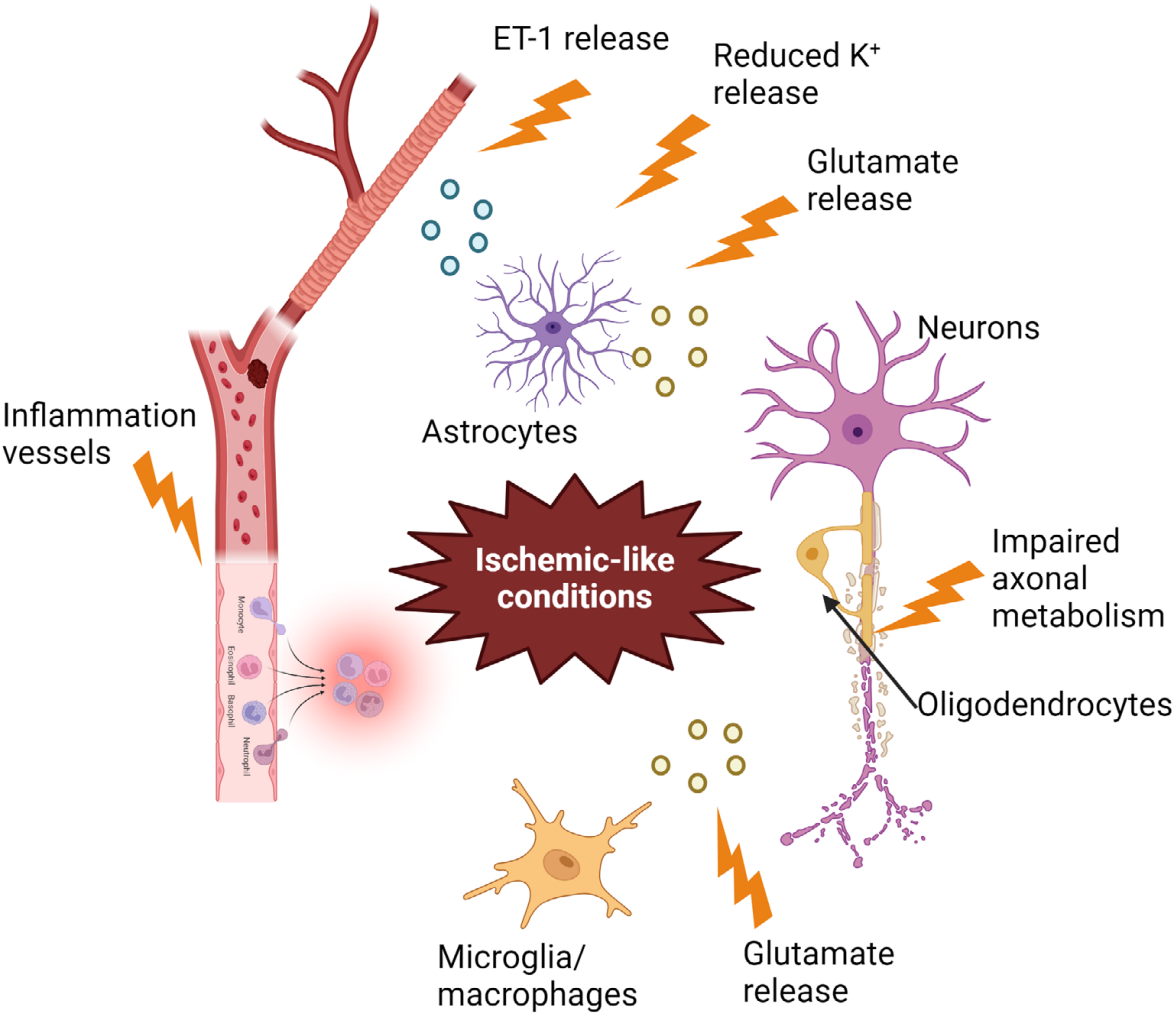
Potential causes of ischemic‐like conditions in MS. Inflammation of the vessel wall may trigger capillary thrombosis and stimulate leukocyte invasion, thereby reducing blood perfusion. Glutamate excitotoxicity may also induce ischemic‐like conditions because of metabolic stimulation. Impaired axonal metabolism may lead to mitochondrial dysfunction and oxidative stress. Abnormal ET‐1 released by astrocytes may cause vasoconstriction. Additionally, reduced K^+^ release by astrocytes, possibly due to decreased expression of β2‐adrenergic receptors may also induce vasoconstriction. Created with BioRender.

Vascularization may be influenced by OPCs, as OPC density correlates with vessel density, and VEGF secreted by OPCs promotes angiogenesis (Zhang et al., 2020). OPCs make contact with sprouting endothelial cells, and the interaction between OPCs and endothelial cells directs white matter vascularization by activating Wnt/β‐catenin signaling in endothelial cells (Chavali et al., 2020; Gao et al., 2023; Pfeiffer et al., 2021; Wang, Pan, et al., 2022). Further, stabilization of HIF1/2α in OPCs induces endothelial cell proliferation, implying a possible role for hypoxia or other factors that cause HIF1/2α stabilization in angiogenesis (Gao et al., 2023; Wang, Pan, et al., 2022; Yuen et al., 2014) (Figure 5).

## 9 CONCLUSION

Metabolic stress is a potential cause of injury to oligodendrocytes and other cells in the CNS. Understanding the structure of the NVU and the communication between the CNS parenchyma and vasculature involved in the regulation of local blood flow is crucial to build an adequate model of the dependence of oligodendrocyte energy requirements on the microenvironment, under physiologic and disease conditions.

Impairments of CNS neurovascular circulation are important components of the etiology of some leukodystrophies, and cerebral hypoperfusion is an early hallmark of MS. Addressing the mechanisms that underlie dysregulation of the microcirculation, BBB integrity, and disruption of the communication between the CNS parenchyma and vasculature provides opportunities to identify targets for treatments to alleviate the impact of hypoperfusion in MS. Furthermore, studying the dynamic relationship between CNS cell types can unveil targets to improve CNS perfusion, with potential benefits for the treatment of demyelinating diseases to promote remyelination and recovery.

## AUTHOR CONTRIBUTIONS

**Milton Guilherme Forestieri Fernandes:** Conceptualization; writing – original draft. **Florian Pernin:** Conceptualization; writing – original draft. **Jack P. Antel:** Conceptualization; writing – review & editing; funding acquisition; supervision. **Timothy E. Kennedy:** Conceptualization; writing – review & editing; funding acquisition; supervision.

### PEER REVIEW

The peer review history for this article is available at https://www.webofscience.com/api/gateway/wos/peer‐review/10.1111/jnc.16219.

## Data Availability

Data sharing is not applicable to this review article as no new data were created or analyzed in this study.
